# Living systematic review and meta-analysis of plasma-concentrations of antipsychotic drugs in carriers and non-carriers of variant CYP450 genotypes: Living systematic review protocol

**DOI:** 10.12688/f1000research.147794.2

**Published:** 2024-07-09

**Authors:** Filip Milosavljević, Stefan Leucht

**Affiliations:** 1 Department of physiology, Faculty of Pharmacy, Univerzitet u Beogradu, Belgrade, 11221, Serbia; 2 Section Evidence-Based Medicine in Psychiatry and Psychotherapy, Department of Psychiatry and Psychotherapy, Klinikum rechts der Isar, TUM School of Medicine and Health, Technical University of Munich, Munich, Bavaria, 81675, Germany

**Keywords:** Living systematic review, Pharmacogenomics, Antipsychotic drugs, Schizophrenia, Therapeutic drug monitoring, CYP450

## Abstract

**Introduction:**

Carriers of variant alleles of genes that encode liver CYP450 and UGT enzymes may experience abnormal plasma levels of antipsychotics and, consequently, worse efficacy or tolerability. Although pharmacogenomics is a rapidly developing field, current guidelines often rely on limited, underpowered evidence. We have previously demonstrated that meta-analysis is a viable strategy for overcoming this problem. Here, we propose a project that will expand our previous work and create a living systematic review and meta-analysis of drug plasma level differences between carriers and non-carriers of variant genotype-predicted phenotypes for every pharmacokinetic drug-gene interaction relevant to commonly used antipsychotic drugs.

**Protocol:**

First, a baseline systematic review and meta-analysis will be conducted by searching for observational pharmacogenomics-pharmacokinetic studies. Data on dose-adjusted drug plasma levels will be extracted, and participants will be grouped based on their genotype for each drug-gene pair separately. Differences in plasma drug levels between different phenotypes will be compared using a random-effect ratio-of-means meta-analysis. The risk of bias will be assessed using ROBINS-I, and the certainty of evidence will be assessed using GRADE. Following the establishment of baseline results, the literature search will be re-run at least once every six months, and the baseline data will be updated and re-evaluated as new evidence is published. A freely available website will be designated to present up-to-date results and conclusions.

**Discussion:**

This systematic review will provide evidence-based results that are continuously updated with evidence as it emerges in the rapidly developing field of pharmacogenomics. These results may help psychiatrists in their decision-making, as clinicians are becoming increasingly aware of the patients’ genetic data as testing becomes more widespread and cheaper. In addition, the results may serve as a scientific basis for the development of evidence-based pharmacogenomics algorithms for personalized dosing of antipsychotics to mitigate potentially harmful drug-gene interactions.

## 1. Introduction

Pharmacotherapy for schizophrenia today is challenging because of (I) slow development of new drugs, ^1^ (II) treatment resistance, ^2^ (III) frequent need for treatment regimen adjustments ^3^ (IV) high relapse rates ^4^ and (V) risk of unpleasant adverse drug reactions. ^4^ In such a landscape, approaches such as pharmacogenomics hold great potential, as many enzymes and transporters involved in the pharmacokinetics of antipsychotics are coded by highly polymorphous genes. ^5^ In theory, mutations of these genes can cause pharmacokinetic, and consequently, pharmacodynamic variability in patients. ^5^^,^^6^ Adjusting drug choice and/or drug dose based on patient’s genomics holds promise to maximize the effectiveness of currently available antipsychotics. ^5^

Pharmacogenomics of pharmacokinetic processes has been researched in the context of phase 1 drug metabolism (oxidation), phase 2 drug metabolism (glucuronization) and drug transport (efflux pumps). ^6^

Phase 1 metabolism is the main mechanism of inactivation for the most of antipsychotics, ^6^ and it predominantly involves CYP450 isoenzymes, namely CYP1A2, CYP2C19, CYP2D6 and CYP3A4/5 ^7^; all of which are coded by highly polymorphic genes. ^8^ CYP450 polymorphism is usually not analyzed in terms of specific genotypes, but rather in terms of genotype combinations called haplotypes or star alleles. The activity of enzymes translated from the specific gene variant is in most cases well characterized in *in-vitro* studies. ^8^ For CYP2C19 and CYP2D6 isoenzymes, consensus is reached on how two star alleles combine into enzyme’s genotype-predicted phenotype; i.e. enzyme’s metabolizer group. ^9^ Four metabolizer groups are commonly defined: Poor metabolizers (PM) – Absent or very low enzyme activity; Intermediate metabolizers (IM) – Significantly reduced enzyme activity; Normal metabolizers (NM; Previously referred to as Extensive metabolizers – EM) – Reference group; and Ultra-rapid metabolizers (UM) – Increased enzyme activity. ^8^ Theoretically, PMs or IMs carry risk of exhibiting too high antipsychotic plasma levels and consequent worse tolerability, ^10^ while UMs carry risk of too low plasma levels and the lack of efficacy. ^10^ Regarding CYP1A2 and CYP3A4/5 isoenzymes, definitions of metabolizer groups based on star allele combinations have been proposed, ^10^ but the broader consensus has not been reached yet. Instead, for CYP1A2 and CYP3A4/5, carriers of each star allele combination are analyzed as a separate group in clinical studies. Besides CYP450 superfamily, few other enzymes coded by polymorphous genes also contribute to the phase 1 metabolism of antipsychotics. For example, AOX1 (Aldehyde oxidase-1) is involved in the metabolism of ziprasidone and FMO3 (Flavin-containing monooxygenase-3) is involved in the metabolism of olanzapine. ^6^

Only few antipsychotics undergo phase 2 metabolism. More specifically, Asenapine, Clozapine, Haloperdiol and Olanzapine all undergo glucuronization. ^6^^,^^11^^,^^12^ This process involves Uridine 5’-diphospho-glucuronosyltransferase (UGT) superfamily, namely UGT1A1, UGT1A3, UGT1A4, UGT1A9, UGT2B7, UGT2B10 izoenzymes ^6^^,^^11^^,^^12^; all of which are coded by polymorphous genes. ^13^ Even though pharmacogenomics of UGT enzymes in psychiatry is understudied, impact of specific genotypes on glucuronization speed has been characterized for other drug classes. ^13^^,^^14^ However, this may pose some issues as there is evidence on substrate specific effect of certain genotypes. ^13^ In theory, genotype-induced variation in glucuronyzation rates can affect plasma concentration of biologically active, unconjugated drugs and thus affect clinical outcomes. ^13^ Even though the definitions of star alleles based on the genotype combination have been proposed, ^14^ majority of clinical trials still report data for each genotype separately, mostly in 3 groups: non-carriers, homozygous carriers and heterozygous carriers of a certain mutation. There is also no consensus on how to define metabolizer groups for UGT enzymes.

Regarding drug transport, many antipsychotics are substrates for the ATP-binding cassette proteins, including multidrug resistance proteins (MDR) coded by ABCB1, ABCC1 and ABCC2 polymorphous genes, and breast cancer resistance protein coded by ABCG2 polymorphous gene. ^6^^,^^15^ Currently, the evidence on the effect of certain genotypes on efflux pump activities is inconsistent and warrants further research. ^15^^,^^16^ Efflux pumps are expressed in many different organs including gastrointestinal tract, kidneys, liver and blood-brain barrier. ^16^ Therefore, genotype-induced changes in efflux pump activities can theoretically impact drug absorption rate, drug elimination rate, drug distribution to the brain, or few of these processes simultaneously. ^16^ Finally, olanzapine and clozapine are substrates to human organic cation transporters hOCT1 and hOCT2 coded by polymorphous SLC22A1 and SLC22A2 genes, respectively. ^11^^,^^12^ These polymorphisms can in theory affect intake of these drugs into hepatocytes ^11^^,^^12^ and affect the proportion of drug available for inactivation.

In order to create an evidence-based pharmacogenomics-informed dosing algorithm that can mitigate these pharmacokinetic drug-gene interactions, their precise extent must be determined first. Despite great volume of research, previous pharmacogenomics-pharmacokinetic studies were mostly inconclusive as there is a common problem of low statistical power. Meta-analysis can be an effective tool for overcoming this problem. ^17^ We plan to build on our previous work on precise quantification of pharmacokinetic drug-gene interactions of antipsychotics ^17^ by including more drug-gene interactions and by utilizing the living systematic review approach.

Since pharmacogenomics is a rapidly developing field and the level of evidence in pharmacogenomics is often unsatisfactory, ^18^^,^^19^ continuously updated evidence-based data sources regarding pharmacokinetic drug-gene interactions could be very informative, as patients’ genomic data is becoming more commonly available to clinicians because commercial pharmacogenomics tools are becoming more widely adopted and cheaper over time.

### 1.1 Research question

Our hypothesis is that polymorphisms in genes encoding CYP450 and/or UGT isoenzymes are associated with significant changes in dose-corrected plasma concentrations of antipsychotic drugs.

Drug-gene pairs will be formed for every relevant polymorphic gene associated with every relevant antipsychotic drug. For every drug gene pair, our aim is to determine, as precisely as possible, the magnitude of the difference in dose-corrected plasma drug concentrations between carriers and non-carriers of variant genotype-predicted phenotypes for the given gene, and the certainty of this evidence. Table 1 presents the PICO format for research questions.

**Table 1.** PICO format for the research question(s). Both general case and two examples of the research question are given. Risperdion-CYP2D6 and Olanzapine-UGT1A4 drug-gene pairs are used as aexamples.

Criterion	General formulation of the research question	Example: Formulation of the research question for the Risperidone-CYP2D6 drug-gene pair	Example: Formulation of the research question for the Olanzapine-UGT1A4 drug-gene pair
P – Population	Adults (18-65 years old) who are either healthy volunteers or psychiatric patients, treated with antipsychotic drug of interest.	Adults (18-65 years old) who are either healthy volunteers of psychiatric patients treated with risperidone.	Adults (18-65 years old) who are either healthy volunteers of psychiatric patients treated with Olanzapine.
I – Intervention(s)	Carrying of a variant genotype-determined phenotype for the gene of interest.	1) Carrying CYP2D6 poor metabolizer phenotype (PM); 2) Carrying CYP2D6 intermediate metabolizer phenotype (IM); 3) Carrying CYP2D6 ultra-rapid metabolizer phenotype (UM).	1) Carrying one UGT1A4*2 allele; 2) Carrying two UGT1A4*2 alleles; 3) Carrying one UGT1A4*3 allele; 4) Carrying two UGT1A4*3 alleles.
C – Control	Carrying of the reference genotype-determined phenotype for the gene of interest.	Carrying CYP2D6 normal metabolizer phenotype (NM).	Carrying UGT1A4*1/*1 alleles.
O – Outcome	Dose-adjusted plasma concentration (C/D) of antipsychotic drug of interest, either after steady state is achieved or after a single drug dose.	Dose-adjusted plasma concentration of risperidone active-moiety (parent drug + active metabolite) either after steady state is achieved or after a single risperidone dose.	Dose-adjusted plasma concentration of olanzapine either after steady state is achieved or after a single olanzapine dose.

## 2. Protocol

This protocol was written in accordance with the PRISMA-P (Preferred reporting items for systematic review and meta-analysis protocols) guideline. ^20^ A checklist is presented in the extended data. The final report of this living systematic review will be written in accordance with MOOSE (Meta-analysis of observational studies) reporting guideline. ^21^ This protocol is also registered with PROSPERO (CRD42024485626).

### 2.1 Literature search

The following databases will be searched: ClinicalTrials.gov, Cochrane Library (Cochrane Database of Systematic Reviews=CDSR and Cochrane Central Register of Controlled Trials=CENTRAL), Embase, MEDLINE, PsycINFO and WHO ICTRP. No studies that were published prior to 1.1.1995. will be searched because of the extremely limited availability of PCR genotyping technology. Both published and unpublished clinical studies will be searched, without language restrictions. The search will be re-run every time the our manuscript is submitted for peer review, as well as continuously once every 6 months during the living meta-analysis maintenance.

Search strategy draft is presented in the extended data.

### 2.2 Drug-gene pairs of interest

Even though many genes can be important for antipsychotic pharmacokinetics, first iteration of our living systematic review will focus on CYP450 and UGT isoenzymes. *AOX-, FMO-, SLC22A-*genes will not be considered at this moment due to the limited amount of research of their pharmacogenomics in context of antipsychotic drugs. Similarly, UGT enzymes other than UGT1A4 and UGT2B7 will not be analyzed in the first iteration of this review due to their relatively limited representation in the literature, and since UGT1A4 and UGT2B7 are among the most abundant UGT isoenzymes in the liver. ^13^

Efflux pump pharamcogenomics will not be considered in this review for two reasons. Firstly, previous research regarding genotype-activity relationships of efflux pumps is inconsistent. ^15^ Secondly, our systematic review presumes that antipsychotic plasma level is a biomarker of its biological activity, and we will assume that cerebrospinal-fluid-to-plasma ratios of antipsychotic drugs are similar between people with different CYP450/UGT genotypes. Since drugs transporters are expressed on blood-brain barrier, it has been demonstrated that changes in efflux pump activity can change cerebrospinal-fluid-to-plasma ratios of antipsychotics. ^22^^,^^23^ This could mean that two study arms with different efflux pump genotypes could have the same antipsychotic drug-plasma levels, but different antipsychotic biological activity. Therefore, we believe that analyzing drug efficacy and safety directly, or analyzing cerebrospinal fluid drug levels are more appropriate outcomes for describing efflux pump pharmacogenomics, all of which are out of the scope of this review.

Based on the information from CPIC, ^6^ PharmVar ^9^ and PharmGKB ^12^ resources, drug-gene pairs of interest were identified and are presented in Table 2. If new relevant data emerges during the maintenance of this living meta-analysis, this list will be updated, and a new drug-gene pair will be included.

**Table 2.** Drug-gene pairs of interest. Drug-gene pairs that will be considered for analysis are formed by pairing most clinically relevant antipsychotic drugs and liver enzymes that are coded by polymorphic genes and are substantially involved in the metabolism of said drugs.

	CYP2D6	CYP3A4/5	CYP2C19	CYP1A2	UGT1A4	UGT2B7
Aripiprazole	X	X				
Asenapine				X	X	
Brexpiprazole	X	X				
Clozapine	X	X	X	X		
Cariprazine	X	X				
Iloperidone	X	X				
Lumateperone		X		X		
Lurasidone		X				
Olanzapine	X	X		X	X	
Quetiapine	X	X				
Risperidone	X	X				
Sertindole	X					
Zotepine		X		X		
Haloperidol	X	X		X		X
Chlorpromazine	X	X		X		
Perphenazine	X			X		
Zuclopentixol	X	X				

Amisulpride and paliperidone will not be analyzed, as they are mainly extracted unchanged via the kidneys. ^6^^,^^24^^,^^25^ Ziprasidone will not be analyzed because of its low inter-individual variability in plasma drug levels. ^26^ First-generation antipsychotic drugs, other than chlorpromazine, perphenazine, haloperidol, and zuclopentixol, were not included because of their limited clinical utility today.

### 2.3 Definition of intervention and control groups

For every drug-gene pair of interest, the exposure group(s) (i.e., variant group; experimental group) and control group (i.e., wild-type group; reference group) will be defined based on the genotype-predicted phenotypes for the gene in question. Detailed definitions of the control and experimental groups based on *CYP2D6, CYP3A4/5, CYP2C19, CYP1A2, UGT1A4* and *UGT2B7* genotypes are adopted from consensus guidelines for every individual gene ^6^^,^^9^^,^^12^ and are presented in detail in the extended data. In short, for *CYP2C19* and *CYP2D6* genes. Normal metabolizer group will be considered as a control, and Intermediate, Poor and Ultra-rapid metabolizer groups will be considered as 3 possible exposure groups. For *CYP1A2*, *CYP3A4*, *UGT1A4* and *UGT2B7* genes, for each variant allele of interest, heterogeneous and homogeneous carriers will be considered as exposure groups while non-carriers will be considered as controls. For *CYP3A5* gene, carriers of two loss-of-function alleles (for example *3/*3) will be considered as exposure group, while every other allele combination will be considered as controls.

It is important to note that, besides genotyping, inter-individual variation in CYP450/UGT activity can also be determined by phenotyping techniques with one or more probe substances. ^27^ Still, genotyping and phenotyping have their own unique advantages and disadvantages. In short, genotyping is error-prone in the case of so-called phenoconversion or other environmental factors, while phenotyping is not. ^27^^,^^28^ Next, phenotyping requires more sophisticated methods and is more resource and time demanding compared to genotyping. ^27^ Finally, genotyping results are not time-dependent are one measurement can be used during entire person’s lifetime while phenotyping results vary over time and require occasional re-analysis. ^28^ Even though both approaches are valid, they cannot be used interchangeably due to abovementioned discrepancies. ^27^ Therefore, we opted to include only genotyping data since it is more compatible with psychiatric clinical practice due to its analytical convenience.

### 2.4 Definition of the outcome

Pharmacokinetic parameters used to represent the drug plasma level will be included only if they are dose adjusted or if the entire cohort is administered the same dose. If necessary and possible, dose-adjusted pharmacokinetic parameters will be manually estimated by dividing the mean drug plasma level and mean drug dose using the Taylor expansion method. ^29^ The potential impact of this estimation will be explored in a sensitivity analysis, where manually estimated dose-adjusted data will be excluded. In addition, for this calculation, a correlation between drug dose and drug plasma levels of ρ=0.5 will be assumed, and two alternative values, ρ=0.2 and ρ=0.8 will be used instead in the sensitivity analysis.

Drug plasma pharmacokinetic parameters are often presented as body-weight-adjusted. To increase the scope of the review, body-weight-adjusted and unadjusted data will be treated as interchangeable. If body-weight-adjusted and unadjusted data are presented simultaneously, body-weight-adjusted data will be prioritized. This decision will be tested using sensitivity analysis, in which only body-weight-adjusted data will be included.

Pharmacokinetic parameters that will be used to assess drug plasma levels will be:

I). Dose-adjusted steady-state drug plasma concentration (Css/D): Measured after five elimination half-lives have elapsed since drug initiation or therapy regimen change, during which patients took stable doses of the drug with good compliance. Ideally, measurements were taken in the morning, just before the new dose was taken.
II). Dose-adjusted area under the steady-state plasma concentration-time curve (AUC/D): Measured after five elimination half-lives had elapsed since drug initiation or therapy regimen change, during which patients took stable doses of the drug with good compliance. Measurements were taken during the same period for all participants.
III). Single-dose plasma concentration (C) or area under the plasma concentration curve (AUC): Even though drug plasma level measurements are not usually considered valid before the steady-state has been achieved owing to unpredictable variability, single-dose pharmacokinetic studies will also be included owing to their high level of confounding control and strict study design. These studies often use homogeneous study groups of healthy volunteers with controlled intake of food, water, and other substances and drugs, with standardized blood sampling times. Thus, well-controlled single-dose pharmacokinetic parameters will be used interchangeably with steady-state parameters, and this decision will be tested in a sensitivity analysis where single-dose studies are excluded. This approach is not valid in the case of drugs with non-linear pharmacokinetic profile, i.e. if drug’s pharmacokinetic parameters are dose-dependent. Even though most of the antipsychotics reviewed here follow linear pharmacokinetics, ^30^ Asenapine, Chlorpromazine, Lumateperone and Perphenazine have been previously detected to have non-linear pharmacokinetic profile. ^31^^–^^34^ Therefore, we will opt to exclude single-dose studies for Asenapine, Chlorpromazine, Lumateperone and Perphenazine. We expect the minor impact of this decision on the final results of our living systematic review due to scarcity of pharmacogenomic data on these specific drugs. Still, if enough single-dose studies are detected for these 4 drugs, we will post-hoc find appropriate mathematical model in the literature in order to approximate steady-state concentrations based on single-dose data.
IV). Reciprocal value of the total drug clearance (1/Cl _total_): For drugs with a linear pharmacokinetic profile, the reciprocal value of the total clearance is equal to the AUC/D metric. If clearance is presented as median with interquartile range and/or minimum and maximum values, these values will be transformed into their reciprocal values, which will be further transformed into mean and standard deviation. ^35^ This decision will be tested in the sensitivity analysis, where reciprocal value data are excluded.

It is important to note that time needed to achieve steady-state may vary between patients and one of the factors influencing this variability is polymorphism in drug metabolizing enzymes. ^36^ Therefore, when elimination half-lives are presented as a range in the AGNP therapeutic drug monitoring guidelines, ^6^ highest limit of this range will be used in order to ensure that experimental groups with slower-than normal drug metabolism have achieved steady-state.

If the blood sample for the Css/D measurement was not taken in the morning before the next dose, but the blood sampling time was consistent in the entire cohort, the study will be included; however, this decision will be tested in the sensitivity analysis where only studies with the ideal sampling times are included.

If the drug possesses one or more active metabolites that are present in the plasma in non-negligible amounts ^6^ compared to the parent drugs, drug plasma levels will be expressed as “Active moiety,” i.e. the sum of the plasma parent drug levels and the plasma active metabolite levels. For risperidone, there is evidence on different blood-brain-barrier permeability of risperidone and its active metabolite 9-OH-risperidone, indicating potential difference in their biological activities of same plasma concentration of these two substances. ^36^ Therefore, data on risperidone/9-OH-risperdione ratio will also be included in the secondary meta-analysis in order to provide better interpretation of risperidone active moiety plasma concentration meta-analysis results.

If multiple pharmacokinetic parameters are present within the same study for the same cohort, Css/D will be prioritized because it is the most common readout in our experience. ^17^ If the same pharmacokinetic parameter is presented for multiple time-points, the longest time point will be selected because of the lower chance that some of the included participants have not yet reached steady-state blood drug levels.

### 2.5 Study inclusion criteria

I). For every drug-gene pair of interest, the study will be included if it reports drug plasma levels for control genotype-predicted phenotype and at least one variant genotype-predicted phenotype for the given gene.
II). Studies involving participants of all ethnicities will be included. However, since the genotype frequencies vary drastically based on ethnic background, subgroup analysis will be performed to compare the results between European, East Asian, South Asian, African, and Admixed American origin cohorts. ^37^
III). Studies in which the participants were treated with antipsychotic drugs were included. This includes studies on patients with schizophrenia, other psychiatric illnesses, and healthy volunteers. To assess the effect of this decision, studies on healthy volunteers will be excluded as a part of the sensitivity analysis.
IV). All age groups were eligible for inclusion in principle, but the main results will be presented only for adults between 18 and 65 years of age. Results on participants of all ages, results only in adolescents (12-18 years old), and the results only for the elderly (>65 years old) will be explored in the sensitivity analysis.
V). Studies will be included regardless of whether the participants were on a stable dosing regimen on enrolment, started drug regimen on enrolment, or switched to a new drug on enrolment.
VI). Studies on both responders and treatment resistant patients will be included.
VII). Studies on participants not exposed to co-medications, foods, or smoking, in cases where these factors are known to interfere with the given antipsychotic drug, are preferred because of the lower risk of confounding. Still, studies where participants are exposed to these confounders will be eligible for inclusion only if these factors are well balanced across different phenotype groups or corrected by the authors during data analysis. In addition, studies with co-medication/food/smoking confounding factors will be excluded as a part of the sensitivity analysis.
VIII). Studies on participants taking any drug formulation (immediate release tablets, extended release tablets, long-acting injections, intravenous administration, etc.) will be eligible, and the data for all formulations will be used interchangeably in the main meta-analysis. To test this decision, every formulation that is not an immediate release tablet will be excluded as a part of the sensitivity analysis.
IX). Prospective studies will be preferred because of the higher reliability of the pre-planned experiments. ^38^ Nevertheless, retrospective studies are very common in pharmacogenomics and will be included to increase the scope of this review. To test this decision, retrospective studies will be excluded as a part of the sensitivity analysis.
X). To increase the scope of this living systematic review, both published and unpublished studies will be included.

### 2.6 Study exclusion criteria

I). Studies on pregnant women will be excluded due to unpredictable pharmacokinetics during pregnancy.
II). Studies with a critical overall risk-of-bias grade based on the ROBINS-I (Risk of bias in non-randomised studies of interventions) tool ^39^ will be excluded (four possible risk-of-bias grades in the ROBINS-I tool are low, moderate, serious, and critical risk of bias, ranked from the best to the worst grade).
III). Studies with a substantial proportion of the population consisting of non-compliers will be excluded because of the risk of erroneous drug plasma level measurements.
IV). Studies on participants with severe kidney or liver impairments or other severe conditions that interfere with drug pharmacokinetics (Malabsorption, Hypoproteinemia, etc.) will be excluded because of unpredictable changes in the pharmacokinetics of these participants.
V). Studies with unbalanced known confounding factors (co-medications, food, cigarette smoke) for drug plasma levels between normal and variant genotype-predicted phenotype groups will be excluded.
VI). Population pharmacokinetic studies will be excluded due to incompatibility with other included studies.

### 2.7 Data screening and selection

Both the screening and selection of the results of the systematic search will be performed independently by two researchers, and any disputes will be resolved by consensus with a third researcher.

Screening will be performed based on the titles and abstracts of the search results. During the screening, search results will be labels as “not relevant,” “relevant” or “unclear relevance.”

For the search results labelled as “relevant” and “unclear relevance,” full text retrieval will be attempted. If the full text could not be retrieved, the corresponding author will be contacted to provide the full text. Next, the retrieved full texts will be assessed for eligibility based on the inclusion and exclusion criteria.

Process of screening and selection will be visually reported using PRISMA 2020 flowchart. ^40^

### 2.8 Data collection and missing data imputation

Data will be extracted using an internally developed standardized form.

Means and standard deviations of drug plasma levels, as well as the number of participants, will be extracted. Means and standard deviations will be estimated using standard procedures if the data are presented as geometric mean and 95%CI, ^41^ as median, interquartile range, minimum, and maximum ^35^ or as mean values and the p-value of the mean difference. ^41^ If the Plasma levels are presented graphically but not numerically, numerical data will be extracted from the digital image of the given graph. ^42^

If patients were not grouped into genotype-predicted phenotypes according to our desired criteria, manual regrouping will be performed when possible. For example, if plasma levels in participants carrying CYP2D6*1/*10 and *1/*5 are presented separately, but there are no data for CYP2D6 IM collectively, the mean plasma levels in *1/*10 and *1/*5 carriers will be summed manually to form the mean IM plasma levels. ^41^

Regarding demographics, data on ethnicity, sex, and age distribution will be collected. Regarding clinical characteristics, data on the participants’ diagnosis, drug dose, body weight, duration of treatment, blood sampling time, co-medications, and other drug-specific confounders will be collected. Finally, data on study design and the characteristics of reported pharmacokinetic parameters will be collected.

If cohorts of two or more clinical studies overlap, inclusion of the studies will be performed to maximize the inclusion of as many unique participants as possible while not having any repeating participants.

If the included manuscript suggests that the data of interest were measured but not presented, the corresponding author will be contacted to provide the data. If this is not possible, the study will be labelled as “Awaiting assessment”. Studies with missing data will be reported as a list and considered during the interpretation of publication bias using funnel plots.

### 2.9 Risk of bias analysis

The ROBINS-I tool ^39^ will be used to assess the seven domains of bias by two researchers independently. Disputes will be resolved by consensus with a third researcher. Based on the semi-quantitative grades for all seven domains, the overall grade for each study will be assigned. Due to the nature of observational studies that will be searched, it is expected for a small number of included studies, if any, to receive the best grade: “Low” risk-of-bias. Instead, it is expected that studies with relatively better design to be graded as having “Moderate” risk-of-bias, while the studies with relatively worse design to receive “Serious” of “Critical” risk-of-bias grade. “No information” overall risk-of-bias grade will be used sparingly, i.e. everywhere where reasonable assumptions regarding the signaling question cannot be made. Studies with a risk of bias grade other than “moderate” or “low” will be excluded as a part of the sensitivity analysis.

### 2.10 Certainty of evidence analysis

Certainty of evidence will be assessed using the GRADE tool ^43^ across five domains of evidence certainty; overall certainty of evidence will also be assessed for every drug-gene pair of interest. GRADE scoring will be performed independently by two researchers, and disputes will be resolved by consensus with a third researcher.

### 2.11 Statistical methods

Quantitative data needed for the meta-analysis includes the mean plasma level, standard deviation, and number of participants in the given study group. For every drug-gene pair analyzed, all experimental groups will be compared pairwise with their corresponding control group. Drug plasma levels will be compared between the groups using the ratio-of-means (RoM) effect measure ^44^ and its 95% confidence interval. This will allow for an intuitive interpretation of the results, as well as the comparison of different pharmacokinetic parameters of drug plasma levels within one analysis. Our previous research showed a high probability of high heterogeneity within the included studies; therefore, a random-effect meta-analysis will be performed. Specifically, RoM=1.15 will be interpreted as 15% higher, and RoM=0.85, which will be interpreted as a 15% lower drug plasma level in the experimental group compared to the control group. RoM values for individual values will be log-transformed and pooled using the inverse-variance method to produce the grand-mean.

As a secondary analysis, all meta-analysis results calculated as RoM will also be calculated as the Standardized Mean Difference (SMD, Hedges’ g). This is aimed at providing an alternative interpretation of the results, which is important considering the rare use of the RoM effect measure.

Heterogeneity will be assessed using the I ^2^ metric, where I ^2^>50% will be considered substantial heterogeneity. ^41^ In the case of substantial heterogeneity, the effect of outlier results on the overall results will be assessed using a sensitivity analysis. Further, heterogeneity will be explored using other sensitivity and subgroup analyses (previously described) to explore what clinical or demographic factors may be the cause of study misalignment.

Potential publication bias and small-study effects will be assessed using contour-enhanced funnel plots ^45^ and Egger’s test. ^46^ If fewer than 10 studies are included for a certain drug-gene pair, publication bias will not be performed, as recommended. ^45^ The critical *p*-value for Egger’s test will be set at p<0.10, as suggested. ^46^

### 2.12 Sensitivity and sub-group analyses

Sensitivity analysis will be used to test the robustness of the obtained results with regard to changes to the inclusion/exclusion criteria. The following sensitivity analyses will be performed: 1) Exclusion of the manually dose-adjustment plasma level data; 2) Usage of ρ=0.2 and ρ=0.8 as an assumed correlations levels between drug dose and drug plasma levels for manual dose-adjustment of plasma levels; 3) Exclusion of body-weight-unadjusted data; 4) Exclusion of the data estimated from reciprocal clearance values; 5) Exclusion of studies with suboptimal time of plasma sampling; 6) Inclusion of data on non-adults; 7) Exclusion of the studies with suboptimal confounding control; 8) Exclusion of “Single drug dose” studies; 9) Exclusion of the studies with “Serious” risk-of-bias overall score; 10) Exclusion of healthy volunteer data; 11) Exclusion of retrospective studies and 12) Exclusion of the data on drug formulations other than immediate release tablets.

Subgroup analyses will be used to compare the meta-analysis results only between participants of different ethnic backgrounds.

### 2.13 Maintenance of the living systematic review and meta-analysis

Following an initial literature search and data analysis, the baseline results and conclusions of the living systematic review and meta-analysis will be established. As these results are being published, designated website will be created to display these results, and thus the maintenance phase of the living systematic review will begin. In this phase, once every six months, a systematic literature search will be repeated, and two designated researchers will be tasked with screening and testing for eligibility of the new search results. Alternatively, if a sudden increase in the number of new publications is detected during the first year of maintenance, updates from that point onwards will be performed more often once every three months. The data from the eligible studies will be extracted in the same manner as during the establishment of the baseline results and will be added to the database of the baseline results. Updated results will then be published on the website, together with the change log. If a new discovery regarding pharmacogenomics of liver enzymes involved in the metabolism of antipsychotics emerges, the entire database will be updated accordingly in the shortest possible notice. This includes newly detected drug-gene pairs of interest and new consensus for genotype-determined phenotype classification.

For pragmatic reasons, once all analyzed drug–gene pairs either achieve satisfactory strength of evidence or experience no new publications for an extended period of time, updates of the living systematic review will be performed less frequently, and the update frequency will depend on the probability of new evidence emerging in the future.

### 2.14 Dissemination

As previously mentioned, the baseline results will be published in separate papers, while the up-to-date results and conclusions will be presented on a designated website. Papers published in scientific journals are planned from time to time during the living systematic review maintenance phase.

### 2.15 Study status

At the time of writing the manuscript (29. January 2024), the literature search strategy was designed, and a systematic search was not performed for either of the drug-genes of interest. No study screening, selection, or data extraction was performed at the time of writing.

## 3. Discussion

This project has the potential to provide much-needed clinical information to the psychiatry community. Psychiatrists are becoming increasingly aware of the CYP450 genetic status of their patients, and the implementation of evidence-based knowledge of drug-gene interactions in psychiatrists’ decision-making will improve the quality of pharmacotherapy in psychiatry. This interaction will be facilitated by up-to-date reporting of the systematic review results on a designated website, which will be freely available to any interested clinician.

Furthermore, several attempts have been made to create and test clinical tools for pharmacogenomics-guided pharmacotherapy for schizophrenia. ^47^^–^^49^ Such tools aim to detect and mitigate potentially dangerous drug-gene interactions, and thus improve the efficacy and/or safety of antipsychotic drugs. One common approach is to completely avoid drugs known to interact negatively with the genetic makeup of a patient. This approach is criticized as potentially dangerous because it can overly restrict the list of effective options available to the patient. ^50^ Another promising approach is to use genetic information to guide the dosing regimen of antipsychotic drugs, but due to limited and often conflicting evidence, genotype-guided dose regimen adjustments are often arbitrary. The results of this systematic review may facilitate the development of new and the improvement of existing pharmacogenomic tools by providing precise estimates of expected blood drug concentration changes in carriers of different genetic variants. This may help in the transition from arbitrary to evidence-based algorithms for the mitigation of potentially harmful drug-gene interactions related to the CYP450 and UGT genotypes.

### 3.1 Limitations

One of the most substantial limitations of this project was the expected high risk of bias in the included studies. This is because of the very common naturalistic approach with insufficient confounder control in the eligible studies observed in our previous work. ^8^ In addition, carriers of variant CYP450 or UGT alleles may be more likely to drop out due to their atypical drug plasma levels during observational studies, potentially biasing the results. Finally, there is a risk that some of the results of this systematic review may become outdated with the discovery of new alleles for some genes of interest. This could cause participants to become misclassified in the studies published prior to this discovery without the option to re-classify them post hoc, as this would require repeated genetic testing.

### Ethics and consent

Ethical approval and written informed consent were not required.

## Data Availability

No data are associated with this article. Figshare, LivingCYP - Living CYP - Extended data - F1000 7jul2024.docx, https://doi.org/10.6084/m9.figshare.25109675.v3 ^51^ Figshare: PRISMA –P checklist for Living systematic review and meta-analysis of plasma-concentrations of antipsychotic drugs in carriers and non-carriers of variant CYP450 genotypes: Living systematic review protocol, https://doi.org/10.6084/m9.figshare.25109675.v3 ^51^ Data are available under the terms of the Creative Commons Attribution 4.0 International license (CC-BY 4.0).
