# Supplementary material for: Living systematic review and meta-analysis of plasma-concentrations of antipsychotic drugs in carriers and non-carriers of variant CYP450 genotypes: Living systematic review protocol
Source: F1000Res. 2024 Jul 9;13:452. Originally published 2024 May 7. [Version 2] doi: 10.12688/f1000research.147794.2 (PMC11292185; doi:10.12688/f1000research.147794.2)
Supplement: Peer review history

# Reviewer report: Reviewer response for version 2

**DOI:** 10.5256/f1000research.168742.r300641

**Published:** 2024-07-31

**Authors:** Pierre Baumann

**Affiliations:** 1 Department of Psychiatry (DP-CHUV), Universite de Lausanne, Lausanne, Vaud, Switzerland

The Introduction is now written more comprehensively, and it is informative. It satisfies the request of the reviewers.

 There are some minor spelling errors (use search function):

 Haloperidol (p. 3, last §)

 I am surprised that drugs (Asenapine, Clozapine, … etc ) and enzymes (Uridine … etc) are (sometimes) written in capital letters (e.g. p. 3 and 7)

 Answer of the Authors to the comment of the reviewer (p. 18): “Even though the pharmacokinetics of inactive drug metabolites is very relevant and important topic, we find this data to be of limited usefulness for our living systematic review as we plan to focus on the clinical aspects of therapeutic drug monitoring.”

 This is somewhat contradictory as this protocol will mainly and almost exclusively focus on the relationship between pharmacokinetics and pharmacogenomics and not on clinical aspects. The determination of ratios parent compound/metabolite (also inactive metabolites) will allow a better understanding of the individual role of a particular enzyme in a specific metabolic pathway. This is only a comment, with no request.

 The reviewer thanks the Authors for their comments and corrections of their manuscript

Is the study design appropriate for the research question?

Partly

Is the rationale for, and objectives of, the study clearly described?

Partly

Are sufficient details of the methods provided to allow replication by others?

Yes

Are the datasets clearly presented in a useable and accessible format?

Not applicable

Reviewer Expertise:

NA

I confirm that I have read this submission and believe that I have an appropriate level of expertise to confirm that it is of an acceptable scientific standard.

# Reviewer report: Reviewer response for version 2

**DOI:** 10.5256/f1000research.168742.r300642

**Published:** 2024-07-16

**Authors:** Jhohann Richard de Lima Benzi

**Affiliations:** 1 University of São Paulo, São Paulo, Brazil

I have reviewed the revised manuscript. The authors have addressed all my comments and questions thoroughly and politely. I have no further comments or concerns. Therefore, I fully endorse this manuscript can be indexed. Thank you for the opportunity to review this work. Best regards.

Is the study design appropriate for the research question?

Partly

Is the rationale for, and objectives of, the study clearly described?

Yes

Are sufficient details of the methods provided to allow replication by others?

Partly

Are the datasets clearly presented in a useable and accessible format?

Yes

Reviewer Expertise:

Clinical pharmacology, pharmacokinetics, pharmacometrics

I confirm that I have read this submission and believe that I have an appropriate level of expertise to confirm that it is of an acceptable scientific standard.

# Reviewer report: Reviewer response for version 1

**DOI:** 10.5256/f1000research.162029.r280401

**Published:** 2024-06-06

**Authors:** Pierre Baumann

**Affiliations:** 1 Department of Psychiatry (DP-CHUV), Universite de Lausanne, Lausanne, Vaud, Switzerland

General comment:

 The authors propose a protocol for future studies on the relationship between drug plasma concentrations in patients treated with antipsychotics and their pharmacogenetic status regarding genetic polymorphisms of cytochrome P-450 isoforms and/or UGT enzymes (cf Table 1, criterion O (outcome)). It would have been of interest to include a part which concerns the clinical outcome (efficacy, effectiveness, adverse effects) of the patients, as the present protocol will not allow to clearly show the clinical relevance of pharmacokinetic-pharmacogenetic exams. However, the present project is already ambitious and the inclusion of a regularly occurring update is highly promising and novel.

 Other comments and questions:

 The issues dealing with pharmacokinetics and pharmacogenomics presented in the Introduction should be presented in more details as some references are not adequate. Indeed, some citations lack precision, e.g. ref 5 and 6, in the sentence: “It is proposed that mutations in genes that code liver CYP450 (Cytochrome p450) and UGT (UDP-glycosyltransferase) enzymes can cause either too low or too high plasma levels of antipsychotic, ^5^ leading to a lack of efficacy or a higher risk of adverse reactions in some patients. ^6^  The citation of reference 5 suggests that it presents data about the importance of UGT in the pharmacogenomics of antipsychotics. However, UGT is only mentioned as a key word (and only as UGT-2B.. but not as UGT-1A..). In ref 6, UGT is not even mentioned. Therefore, the reader with limited knowledge in this field may not get a full picture of the importance of the subject. In summary, the Introduction should be extended!

 Table 1 presents in “Intervention I” the different phenotypes using the example risperidone. The reader is then supposed to presume that this presentation is also valid for UGT enzymes (eg gene-drug pair: UGT-olanzapine), but this classification (poor, intermediate, extensive, ultrarapid) is seemingly not introduced for these polymorphisms. If however this is the case, this should be presented in some details and corresponding references should be presented.

 Indeed, the average reader will not be familiar with the genetic polymorphisms of conjugating enzymes, and in particular, with their relevance for antipsychotics. In other words: are there PM, EM, UM, IM in patients treated with e.g. asenapine or olanzapine, with regard to the metabolism by UGT-enzymes? (Admittedly, in the suppl. material is some information, but the protocol should be more precise, especially in Introduction). It is a fact that among cytochrome P-450 isozymes, only about half a dozen isozymes play a role in the metabolism of antipsychotics. In contrast, there are considerably more isozymes which contribute to the conjugation of drugs, but their individual role in the metabolism of antipsychotics is poorly characterized.

 The pioneering study of Haslemo et al. (CPT, 2012, 221) (besides that of Erickson et al., Pharmacogen Genom 2011, 539) shows that plasma concentrations of conjugated olanzapine (10-N-ola-glucuronide) differ in olanzapine treated patients classified according to their UGT1A genotypes, but the difference is negligible for (unconjugated) olanzapine concentrations. This rises the question whether in the present meta-analysis study both ola and conjugated ola concentrations will be considered. It could be then of interest to examine the relationships between the UGT genotypes and the ratios parent compound/metabolite (Ola/10-N-ola-glucoronide).

 This approach may also be valuable for all other antipsychotics, whether they are metabolized by cytochrome P-450 or by polymorphic conjugating enzymes, or by both types (e.g. risp/9-OH-risp; 9-OH-risp/conjug. 9-OH-risp; etc).

 Probably of little importance for the establishment of the protocol, it is to mention that in the case of risperidone, its hydroxylation to 9-OH-risperidone is stereoselective, in that CYP2D6 preferentially forms (+)-9-OH-risperidone rather than (-)-9-OH-risperidone (Yasui-Furukori et al. Drug Metab. Disp 2001, 1263). It should be considered, as rarely, stereoselective procedures were used in the published studies, the net effect of the genotype may be masked when non-stereoselective procedures are used.

 The authors exclude some antipsychotics, but the mentioned reasons are not always evident, and again, the citations do not always correspond to the statements. Therefore, the presentation of the reference list demands to be improved. Eg, 2.2. Drug gene pairs …:

 “Lurasidone will not be analyzed because of the uncertain importance of drug-blood level measurements.” Ref 6 is cited, but this ref does apparently not deal with this subject. Actually, lurasidone is mainly a substrate of CYP3A4/5. As its blood levels strongly depend on dietary conditions, this may be an important reason for recommending TDM (cf Preskorn 2013: Lurasidone should be administered with food-at least 350 kcal-to ensure maximum exposure). However, one can agree with the decision of the authors, as this drug belongs to the group of drugs mentioned in 2.5. VII (study inclusion citeria) resp. 2.6. V.

 2.4. Definition of the outcome

 As pointed out by the authors, weight corrected drug concentrations may be advantageous, but they are often not available.

 Steady-state plasma concentrations: In the case of risperidone, there may be a problem in that in extensive metabolizers (CYP2D6), its T1/2 = 2 - 4h, but in poor metabolizers, considerably longer, comparable to that of its metabolite 9-OH-risperidone. This means that in EM, risp. steady-state concentrations will hardly be reached in EM but only in PM.

 Extended data: March :

**6. UGT1A4**

 “At the time of writing, there is no consensus regarding the classification of different UGT1A4 diplotypes into metabolizer groups. Therefore, pragmatically, alleles known to affect CYP1A2 activity with Minor allelic frequencies >1% in the world population will be considered for this analysis.” Not clear: this suggests that there is a relationship CYP1A2 – UGT1A4 (?).

Is the study design appropriate for the research question?

Partly

Is the rationale for, and objectives of, the study clearly described?

Partly

Are sufficient details of the methods provided to allow replication by others?

Yes

Are the datasets clearly presented in a useable and accessible format?

Not applicable

Reviewer Expertise:

NA

I confirm that I have read this submission and believe that I have an appropriate level of expertise to confirm that it is of an acceptable scientific standard, however I have significant reservations, as outlined above.

# Response

**Published:** 2024-07-01

**Authors:** Filip Milosavljević

**Affiliations:** EBMpp, Technical University of Munich, Munich, Bavaria, Germany

*"The authors propose a protocol for future studies on the relationship between drug plasma concentrations in patients treated with antipsychotics and their pharmacogenetic status regarding genetic polymorphisms of cytochrome P-450 isoforms and/or UGT enzymes (cf Table 1, criterion O (outcome)). It would have been of interest to include a part which concerns the clinical outcome (efficacy, effectiveness, adverse effects) of the patients, as the present protocol will not allow to clearly show the clinical relevance of pharmacokinetic-pharmacogenetic exams. However, the present project is already ambitious and the inclusion of a regularly occurring update is highly promising and novel."*

 We thank the reviewer for positive comment and for recognizing the scale of this project.

*"Other comments and questions:*

*The issues dealing with pharmacokinetics and pharmacogenomics presented in the Introduction should be presented in more details as some references are not adequate. Indeed, some citations lack precision, e.g. ref 5 and 6, in the sentence: “It is proposed that mutations in genes that code liver CYP450 (Cytochrome p450) and UGT (UDP-glycosyltransferase) enzymes can cause either too low or too high plasma levels of antipsychotic,5 leading to a lack of efficacy or a higher risk of adverse reactions in some patients.6  The citation of reference 5 suggests that it presents data about the importance of UGT in the pharmacogenomics of antipsychotics. However, UGT is only mentioned as a key word (and only as UGT-2B.. but not as UGT-1A..). In ref 6, UGT is not even mentioned. Therefore, the reader with limited knowledge in this field may not get a full picture of the importance of the subject. In summary, the Introduction should be extended!"*

 We sincerely apologize for inaccurate citations. In accordance with this comment and the comment from the other reviewer, introduction section is now expended to shortly summarize all gene candidates potentially important for the pharmacogenomics of antipsychotic drug pharmacokinetics.

*"Table 1 presents in “Intervention I” the different phenotypes using the example risperidone. The reader is then supposed to presume that this presentation is also valid for UGT enzymes (eg gene-drug pair: UGT-olanzapine), but this classification (poor, intermediate, extensive, ultrarapid) is seemingly not introduced for these polymorphisms. If however this is the case, this should be presented in some details and corresponding references should be presented."*

 We agree, Table 1 can be misinterpreted. In the introduction section, it is now described which genes have established metabolizer status definitions and which genes are instead analyzed on the basis of genotypes and haplotypes. Also, Table 1 is now updated to include another example that involves UGT isoenzyme to further eliminate confusion.

*"Indeed, the average reader will not be familiar with the genetic polymorphisms of conjugating enzymes, and in particular, with their relevance for antipsychotics. In other words: are there PM, EM, UM, IM in patients treated with e.g. asenapine or olanzapine, with regard to the metabolism by UGT-enzymes? (Admittedly, in the suppl. material is some information, but the protocol should be more precise, especially in Introduction). It is a fact that among cytochrome P-450 isozymes, only about half a dozen isozymes play a role in the metabolism of antipsychotics. In contrast, there are considerably more isozymes which contribute to the conjugation of drugs, but their individual role in the metabolism of antipsychotics is poorly characterized."*

 In line with this and previous comments, introduction section is now extended to also include this information. Also, our justification for the choice of UGT isoenzymes included in the first iteration of our living systematic review is included in the section 2.2. If the Reviewer finds that some crucial UGT or CYP450 isoenzymes are omitted, we will gladly consider them for the inclusion.

 "The pioneering study of Haslemo et al. (CPT, 2012, 221) (besides that of Erickson et al., Pharmacogen Genom 2011, 539) shows that plasma concentrations of conjugated olanzapine (10-N-ola-glucuronide) differ in olanzapine treated patients classified according to their UGT1A genotypes, but the difference is negligible for (unconjugated) olanzapine concentrations. This rises the question whether in the present meta-analysis study both ola and conjugated ola concentrations will be considered. It could be then of interest to examine the relationships between the UGT genotypes and the ratios parent compound/metabolite (Ola/10-N-ola-glucoronide).This approach may also be valuable for all other antipsychotics, whether they are metabolized by cytochrome P-450 or by polymorphic conjugating enzymes, or by both types (e.g. risp/9-OH-risp; 9-OH-risp/conjug. 9-OH-risp; etc)."

 To our knowledge, Olanzapine metabolites including 10-N-Olanzapine-Glucuronide have marginal activity and it is not recommended to measure them during therapeutic drug monitoring [PMID: 1683407]. Even though the pharmacokinetics of inactive drug metabolites is very relevant and important topic, we find this data to be of limited usefulness for our living systematic review as we plan to focus on the clinical aspects of therapeutic drug monitoring.

 Regarding risperidone, there is evidence that risperidone and 9-OH-risperidone have different efficacy and safety profiles due to different blood-brain-barriers permeability [PMID: 33054667]. Therefore, we agree for that analysis of riperidone/9-OH-risperidone ratio may be very useful for the interpretation of meta-analysis of risperidone active moiety (Risperidone + 9-OH-Risperidone) plasma levels.

 Section 2.4 is now updated to reflect this.

*"Probably of little importance for the establishment of the protocol, it is to mention that in the case of risperidone, its hydroxylation to 9-OH-risperidone is stereoselective, in that CYP2D6 preferentially forms (+)-9-OH-risperidone rather than (-)-9-OH-risperidone (Yasui-Furukori et al. Drug Metab. Disp 2001, 1263). It should be considered, as rarely, stereoselective procedures were used in the published studies, the net effect of the genotype may be masked when non-stereoselective procedures are used."*

 As we mentioned previously, we would like to focus on clinical aspects of therapeutic drug monitoring, i.e. we will try to use drug blood levels as a biomarkers of drugs biological effects. Therefore, even though stereo-selective analysis highlights the influence of CYP2D6, we find very limited clinical relevance of this information for our review as two enantiomers of 9-OH-Risperidone have been previously described as equipotent (PMID: 17344104).

*"The authors exclude some antipsychotics, but the mentioned reasons are not always evident, and again, the citations do not always correspond to the statements. Therefore, the presentation of the reference list demands to be improved. Eg, 2.2. Drug gene pairs …:*

*“Lurasidone will not be analyzed because of the uncertain importance of drug-blood level measurements.” Ref 6 is cited, but this ref does apparently not deal with this subject. Actually, lurasidone is mainly a substrate of CYP3A4/5. As its blood levels strongly depend on dietary conditions, this may be an important reason for recommending TDM (cf Preskorn 2013: Lurasidone should be administered with food-at least 350 kcal-to ensure maximum exposure). However, one can agree with the decision of the authors, as this drug belongs to the group of drugs mentioned in 2.5. VII (study inclusion citeria) resp. 2.6. V."*

 We agree with the reviewer, Lurasidone is the substrate for CYP3A4/5 and should therefore be included. Also, unrelated to reviewer’s comment, we have noticed that we listed Paliperidone as CYP3A4/5 and CYP2D6 substrate even though paliperidone is minimally metabolized in the liver and is mostly eliminated by kidneys [PMID: 20118446].

 We sincerely apologize for these errors; they are now corrected in the revised version of the manuscript.

*"2.4. Definition of the outcome*

*As pointed out by the authors, weight corrected drug concentrations may be advantageous, but they are often not available.*

*Steady-state plasma concentrations: In the case of risperidone, there may be a problem in that in extensive metabolizers (CYP2D6), its T1/2 = 2 - 4h, but in poor metabolizers, considerably longer, comparable to that of its metabolite 9-OH-risperidone. This means that in EM, risp. steady-state concentrations will hardly be reached in EM but only in PM."*

 We agree, people with lower enzyme metabolism capacities might take longer time to reach steady state. Therefore, we changed the criterion for the time needed to reach steady state to be 5x the conservative t _1/2 _estimate. In other words, whenever the t _1/2_ is presented as the range in AGNP guidelines (PMID: 28910830) we will use the higher limit of this range (longest time). This is now reflected in section 2.4.

*"Extended data: March :*

*6. UGT1A4*

*“At the time of writing, there is no consensus regarding the classification of different UGT1A4 diplotypes into metabolizer groups. Therefore, pragmatically, alleles known to affect CYP1A2 activity with Minor allelic frequencies >1% in the world population will be considered for this analysis.” Not clear: this suggests that there is a relationship CYP1A2 – UGT1A4 (?)."*

 This was a typing error, and we sincerely apologize for it. We also noticed several other typing errors in the Extended data and they are now corrected in the new version.

 We again thank the Reviewer for the constructive criticism. We have no further comments.

# Reviewer report: Reviewer response for version 1

**DOI:** 10.5256/f1000research.162029.r275387

**Published:** 2024-05-31

**Authors:** Jhohann Richard de Lima Benzi

**Affiliations:** 1 University of São Paulo, São Paulo, Brazil

The manuscript under review presents a comprehensive systematic review protocol into the relevance of CYP pharmacogenetics in antipsychotic (AP) therapy, offering a clear and well-written discourse on the topic. The study's goal is undeniably pertinent, addressing a critical aspect of personalized medicine in psychiatric care. I commend the authors for their meticulous approach and the significance of their endeavors. However, I would like to suggest some considerations to enrich the manuscript.

 Firstly, the inclusion or mention of the importance of drug transporters in AP therapy warrants attention, particularly those expressed in the blood-brain barrier. Given their pivotal role in drug disposition and response, their omission from the review raises questions about their relevance in AP pharmacotherapy. Exploring the available pharmacogenetic data pertaining to these transporters could provide valuable insights into their impact on treatment outcomes.

 Secondly, the decision to exclude data based on probe phenotyping drug data when defining metabolizer groups requires clarification. Elaborating on the rationale behind this exclusion would enhance the transparency and reproducibility of the study, ensuring a more robust analysis of CYP pharmacogenetics in AP therapy.

 Thirdly, I respectfully disagree with the assertion that single-dose pharmacokinetic parameters can be used interchangeably with steady-state parameters, particularly in the context of AP drugs with non-linear pharmacokinetics. Given the complexities inherent in non-linear PK profiles, a sensitivity analysis excluding single-dose studies may not adequately capture the dynamic nature of drug metabolism. It is imperative to devise alternative strategies to handle such data, perhaps through modeling techniques or subgroup analyses, to ensure the integrity and reliability of the findings.

 In conclusion, while the manuscript admirably addresses the relevance of CYP pharmacogenetics in AP therapy, the incorporation of drug transporter considerations, clarification on data exclusion criteria, and careful handling of non-linear PK data are essential for a more comprehensive and nuanced analysis.

Is the study design appropriate for the research question?

Partly

Is the rationale for, and objectives of, the study clearly described?

Yes

Are sufficient details of the methods provided to allow replication by others?

Partly

Are the datasets clearly presented in a useable and accessible format?

Yes

Reviewer Expertise:

Clinical pharmacology, pharmacokinetics, pharmacometrics

I confirm that I have read this submission and believe that I have an appropriate level of expertise to confirm that it is of an acceptable scientific standard, however I have significant reservations, as outlined above.

# Response

**Published:** 2024-07-01

**Authors:** Filip Milosavljević

**Affiliations:** EBMpp, Technical University of Munich, Munich, Bavaria, Germany

*"The manuscript under review presents a comprehensive systematic review protocol into the relevance of CYP pharmacogenetics in antipsychotic (AP) therapy, offering a clear and well-written discourse on the topic. The study's goal is undeniably pertinent, addressing a critical aspect of personalized medicine in psychiatric care. I commend the authors for their meticulous approach and the significance of their endeavors. However, I would like to suggest some considerations to enrich the manuscript."*

 We thank the reviewer for positive feedback.

*"Firstly, the inclusion or mention of the importance of drug transporters in AP therapy warrants attention, particularly those expressed in the blood-brain barrier. Given their pivotal role in drug disposition and response, their omission from the review raises questions about their relevance in AP pharmacotherapy. Exploring the available pharmacogenetic data pertaining to these transporters could provide valuable insights into their impact on treatment outcomes."*

 Indeed, drug efflux pumps are coded by highly polymorphous genes (ABCB1, ABCG2, ABCC2…) and the research of their pharmacogenomics has been very active in the recent years. We initially omitted to mention drug transporter in an effort to keep our report concise, but we agree with the reviewer that these genes have to be addressed in order to provide comprehensive overview of pharmacogenomics of antipsychotic drug pharmacokinetics, and that the omission of drug transporter genes from our analysis has to be justified in the manuscript.

 The main reason to exnclude pharmacogenomic studies on efflux pumps from our review is potential inappropriateness of our main outcome to capture the impact of pharmacogenomics of drug transporters on antipsychotic therapy. Our review is focused on plasma levels of antipsychotics, and in our analysis we will assume that cerebrospinal-fluid-to-plasma ratios of antipsychotic drugs are similar between people with different CYP450/UGT genotypes. Since drugs transporters are expressed on blood-brain barrier, in some antipsychotic drugs it has been demonstrated that mutations of drug transporter genes such as ABCB1 can change cerebrospinal-fluid-to-plasma ratios [PMID: 21148022, PMID: 20599439, PMID: 24666334]. This means that, for example, if two groups of patients with different ABCB1 mutations have the same antipsychotic plasma levels, they may not have the same antipsychotic cerebrospinal fluid levels, which puts into question the appropriateness of drug plasma level as a biomarker. Therefore, we believe that analyzing drug efficacy and safety directly, or analyzing cerebrospinal fluid drug levels are more appropriate outcomes for the analysis of drug transporters pharmacogenomics, all of which are out of the scope of this review.

 Introduction section now includes short summary of the potential significance of efflux pumps, and Section 2.2. now includes the justification of our justification not to include efflux pump pharmacogenomic data.

*"Secondly, the decision to exclude data based on probe phenotyping drug data when defining metabolizer groups requires clarification. Elaborating on the rationale behind this exclusion would enhance the transparency and reproducibility of the study, ensuring a more robust analysis of CYP pharmacogenetics in AP therapy."*

 We agree with the reviewer that this decision needs more clarification. Also, upon revision of our manuscript, we noticed that our previous wording “for the purpose of this review, phenotyping will not be considered valid” can be interpreted as us questioning the validity of phenotyping method, which is not the case. Instead, section 2.3 is now updated to introduce pros and cons of both phenotyping and genotyping approaches and the justification why we opted not to use them interchangeably in this review.

*"Thirdly, I respectfully disagree with the assertion that single-dose pharmacokinetic parameters can be used interchangeably with steady-state parameters, particularly in the context of AP drugs with non-linear pharmacokinetics. Given the complexities inherent in non-linear PK profiles, a sensitivity analysis excluding single-dose studies may not adequately capture the dynamic nature of drug metabolism. It is imperative to devise alternative strategies to handle such data, perhaps through modeling techniques or subgroup analyses, to ensure the integrity and reliability of the findings."*

 We thank the reviewer for raising this concern. Even though the majority of antipsychotics have linear pharmacokinetic profile, sublingual asenapine, oral lumateperone, oral chlorpromazine and oral perphenazine possess non-linear pharmacokinetic profiles (PMID: 29915922, PMID: 32060882, PMID: 8157044, PMID: 3933033). We agree with the reviewer that for the given examples, data from single dose studies and steady state studies cannot be used interchangeably and we apologize for our omission. As we are currently not aware of any model published in the literature designed to predict steady state levels of given four drugs based on single-dose data, we will, for the time being, opt to exclude single-dose studies for asenapine, lumateperone, chlorpromazine and perphenazine. We expect very small (if any) impact of this decision on the results of our meta-analyses, as we, based on our previous experience, expect to detect very few pharamcogenomic studies in the literature on these 4 drugs. If instead we happen to detect many eligible single-dose studies for these 4 drugs, we will post-hoc find appropriate model developed for other drugs that may fit the drug in question.

 This clarification is now reflected in the manuscript.

*"In conclusion, while the manuscript admirably addresses the relevance of CYP pharmacogenetics in AP therapy, the incorporation of drug transporter considerations, clarification on data exclusion criteria, and careful handling of non-linear PK data are essential for a more comprehensive and nuanced analysis."*

 We thank the reviewer again for the constructive criticism that will improve the quality of this paper. We have no further comments.
